# FlpS, the FNR-Like Protein of *Streptococcus suis* Is an Essential, Oxygen-Sensing Activator of the Arginine Deiminase System

**DOI:** 10.3390/pathogens5030051

**Published:** 2016-07-21

**Authors:** Jörg Willenborg, Anna Koczula, Marcus Fulde, Astrid de Greeff, Andreas Beineke, Wolfgang Eisenreich, Claudia Huber, Maren Seitz, Peter Valentin-Weigand, Ralph Goethe

**Affiliations:** 1 Institute for Microbiology, University of Veterinary Medicine Hannover, 30173 Hannover, Germany; joerg.willenborg@tiho-hannover.de (J.W.); anna.koczula@tiho-hannover.de (A.K.); marcus.fulde@fu-berlin.de (M.F.); maren.seitz@tiho-hannover.de (M.S.); peter.valentin@tiho-hannover.de (P.V.-W.); 2 Animal Sciences Group (ASG) of Wageningen UR, 8219 PH Lelystad, The Netherlands; Astrid.degreeff@wur.nl; 3 Institute for Pathology, University of Veterinary Medicine Hannover, 30173 Hannover, Germany; andreas.beineke@tiho-hannover.de; 4 Lehrstuhl für Biochemie, Technische Universität München, 85748 Garching, Germany; wolfgang.eisenreich@mytum.de (W.E.); claudia.huber@mytum.de (C.H.)

**Keywords:** *Streptococcus suis*, FNR-like protein, arginine deiminase system

## Abstract

*Streptococcus* (*S.*) *suis* is a zoonotic pathogen causing septicemia and meningitis in pigs and humans. During infection *S. suis* must metabolically adapt to extremely diverse environments of the host. CcpA and the FNR family of bacterial transcriptional regulators are important for metabolic gene regulation in various bacteria. The role of CcpA in *S. suis* is well defined, but the function of the FNR-like protein of *S. suis*, FlpS, is yet unknown. Transcriptome analyses of wild-type *S. suis* and a *flpS* mutant strain suggested that FlpS is involved in the regulation of the central carbon, arginine degradation and nucleotide metabolism. However, isotopologue profiling revealed no substantial changes in the core carbon and amino acid de novo biosynthesis. FlpS was essential for the induction of the *arcABC* operon of the arginine degrading pathway under aerobic and anaerobic conditions. The *arcABC*-inducing activity of FlpS could be associated with the level of free oxygen in the culture medium. FlpS was necessary for *arcABC*-dependent intracellular bacterial survival but redundant in a mice infection model. Based on these results, we propose that the core function of *S. suis* FlpS is the oxygen-dependent activation of the arginine deiminase system.

## 1. Introduction

*Streptococcus suis* is one of the most important porcine pathogens causing meningitis, septicemia, and polyarthritis. *S. suis* infections lead to high financial losses in the swine-producing industry worldwide [1]. In addition, *S. suis* is considered a neglected zoonotic pathogen that can cause pathologies such as meningitis and septicemia, as well as a streptococcal toxic shock-like syndrome in humans [2,3,4,5].

Little is known about the pathogenicity of *S. suis* in porcine as well as human infections. Besides, what is known about the antiphagocytic polysaccharide capsule and several proposed virulence-associated factors [6], elucidation of global regulatory networks that coordinate an efficient adaptation to changing metabolic and environmental stimuli, is a prerequisite for understanding the pathogenesis of *S. suis* infections.

The CRP/FNR family consists of a group of auto-regulated, one-component transcriptional regulators with a conserved C-terminal helix-turn-helix domain responsible for DNA binding. The heterogeneous N-terminus seems to sense a broad spectrum of metabolic co-factors, e.g., cAMP, oxygen, nitrogen, or heme [7,8]. In Gram-negative bacteria, the CRP/FNR-type regulator family was first described as homologous transcriptional regulator proteins with different functions. While CRP (cAMP receptor protein) was the first protein shown to be crucial for the control of catabolite repression [9], FNR (regulator for fumarate and nitrate reduction) has been shown to be involved in oxygen-regulated gene expression [7,8]. In an oxygen-rich environment, FNR proteins are inactive monomers unable to bind regulatory DNA sequences. However, when oxygen becomes limited, FNR proteins can interact via four N-terminal cysteine residues with [4Fe ± 4S] clusters to form homodimers and bind to DNA, leading to transcription activation or repression at specific target promoters [10,11,12]. FNR-like proteins of Gram-positive bacteria often contain only two cysteine residues instead of four as described for Gram-negative bacteria [13,14,15], and their activation by [4Fe ± 4S] cluster formation is controversially discussed.

The arginine deiminase system (ADS) represents a metabolic pathway of arginine degradation which provides energy, CO_2_, and nitrogen in the form of ammonia. It is widely distributed among Gram-positive and Gram-negative bacteria [13,16,17,18,19,20,21,22]. ADS activity has been found to be regulated by several environmental stimuli [23]. In many bacteria, *arcABC* expression is induced under anaerobic conditions [13,21,24,25,26]. It is suggested that the *arcABC* expression might be involved in anaerobic energy metabolism by providing a route for anaerobic substrate-level phosphorylation [27].

The genes encoding for the arginine-degrading activity of the ADS of *S. suis* are transcribed from a single mRNA, the *arcABC* operon, which is followed by the *arcD* gene, encoding a membrane-bound arginine-ornithine antiporter, as well as a putative aminopeptidase-encoding *arcT* gene and the arginine regulator-encoding *argR* gene. The *arcABC* operon encodes for the key enzymes, arginine deiminase, ornithine carbamoyl-transferase, and carbamate kinase, which catalyze the conversion of arginine to ornithine, thereby producing ammonia, carbon dioxide and one mole of ATP per mole of arginine [28,29]. For *S. suis* the *arcABC* operon ensures optimal bacterial growth, and the production of ammonia and ATP provides energy and protection of *S. suis* against acid stress [30,31,32].

In our previous work we demonstrated that ArgR is essential for the induction of the ADS and thereby contributes to the biological fitness of *S. suis* in epithelial cells [33]. However, the expression levels of the *arcABC* operon of *S. suis* are highly dependent on different environmental stimuli such as the glucose level, arginine concentration and anaerobic conditions [23]. Importantly, also in other bacteria, sensing of these stimuli and subsequent *arcABC* regulation depend on the activity of specific conserved metabolic transcriptional regulators. Though the representation of those regulators is conserved among different bacterial species, their function in terms of *arcABC* regulation might vary tremendously. For example, glucose-induced carbon catabolite repression (CCR) in *S. suis* is indirectly [34] and, in contrast to *S. gordonii*, not predominately mediated by the catabolite control protein A (CcpA) [13,35]. Similar to other bacteria, *arcABC* expression positively correlates with concentrations of free arginine in the culture medium; however, the function of ArgR is limited to *arcABC* operon regulation.

FNR and FNR-like proteins are known to be involved in ADS regulation. In *Pseudomonas aeruginosa* and *Staphylococcus aureus* they mediate the oxygen-dependent activation of the ADS [21,24,26], and in *S. gordonii arcABC* expression was linked to the activity of an FNR-like protein, Flp [13]. In *Escherichia (E.) coli* FNR and CRP can regulate the expression of overlapping target genes in response to environmental (oxygen/redox) or metabolic (cAMP) stimuli, [14]. CcpA and the FNR-like regulators are also important for metabolic gene regulation in Gram-positive bacteria. In *S. suis* the role of CcpA is well defined, but the function of the FNR-like regulator FlpS is yet unknown. The predicted gene for the FNR-like protein of *S. suis*, FlpS, is located directly upstream of the *arcABC* operon [23], suggesting a role of FlpS in ADS regulation. In the present study, using growth experiments, transcriptome and gene expression analyses, isotopologue profiling, and mice infection models, we investigated the role of FlpS in a serotype 2 strain of *S. suis* in more detail.

## 2. Results

### 2.1. FlpS is Involved in the Regulation of the *arcABC* Operon and Metabolic Genes

**Figure 1:**
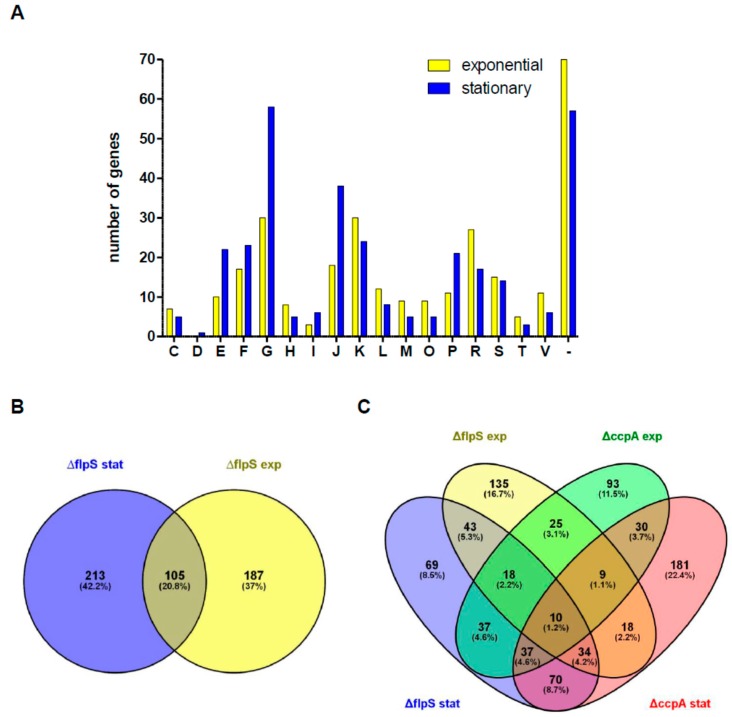
Influence of FlpS knock-out on global gene expression during growth of *S. suis*. (**A**) Summary of significantly differentially expressed genes during exp and stat growth of *S. suis* strain 10Δ*flpS* and classification of clusters of orthologous groups (COG). C, energy production and conversion; D, cell cycle control, cell division; E, amino acid transport and metabolism; F, nucleotide transport and metabolism; G, carbohydrate transport and metabolism; H, coenzyme transport and metabolism; I, lipid transport and metabolism; J, translation; K, transcription; L, replication, recombination and repair; M, cell wall/membrane biogenesis; O, post-translational modification, protein turnover, chaperones; P, inorganic ion transport and metabolism; R, general function prediction only; S, function unknown; T, signal transduction mechanisms; V, defense mechanisms; [−], no prediction; (**B**) Venn diagram illustration of the number of significant differentially expressed genes during exp and stat growth of *S. suis* strain 10Δ*flpS*; (**C**) Venn diagram illustration of the number of significant differentially expressed genes during exp and stat growth of *S. suis* strains 10Δ*flpS* and 10Δ*ccpA* [34].

In order to dissect the role of FlpS in *S. suis*, we first determined the FlpS regulon. For this, we generated a *flpS* knock-out strain (10Δ*flpS*) by insertion mutagenesis. The functional knock-out of the *flpS* gene was controlled by Northern blot analysis (Figure S1B). Following this, we compared the transcriptome of strain 10Δ*flpS* and its parental wild-type strain 10 grown to the exponential (exp) or to the early stationary (stat) growth phase (Figure S1A) by cDNA microarray analyses. Microarray data were additionally validated by RT-qPCR experiments for a selected subset of genes in the wild-type strain 10 and strain 10Δ*flpS* (Table S2). Overall, 292 and 318 genes were found to be differentially expressed in strain 10Δ*flpS* in the exp and stat growth phase, respectively (Figure 1B). In the exp growth phase, higher expression of 208 genes and lower expression of 84 genes suggested both a repressing and activating regulatory role for FlpS. In the stat growth phase this distribution changed to 172 higher-expressed and 142 lower-expressed genes. Classification of clusters of orthologous groups (COG) indicated that, apart from genes that could not be assigned to any specific function, the majority of differentially expressed genes in both growth phases of strain 10Δ*flpS* were associated with carbohydrate transport/metabolism, transcriptional regulation, translation and nucleotide transport/metabolism (Figure 1A, Tables S3–S5).

The *flpS* knock-out markedly affected the expression of the arginine deiminase operon (*arcABC*). Thus, it was approximately 150-fold lower-expressed in the stat growth phase when compared to the wild-type strain, indicating that FlpS is an essential activator of *arcABC* expression. The influence of FlpS on the expression of the other differentially expressed genes was considerably lower, which suggests a less dominant role of FlpS in their regulation.

We have previously shown that the regulator ArgR is an essential, system-specific regulator of the *arcABC* operon [33]. Thus, the *argR* knock-out specifically affected *arcABC* operon expression without influencing the expression of any other gene. This finding suggested a functional relevance of the weak but differentially regulated genes in strain 10Δ*flpS*. Thus, we analyzed these gene subsets in more detail. Among those, several genes encoded for PTS systems for the uptake of alternative carbohydrate sources. In the exp growth phase, PTS for maltose (*SSU0395*) and lactose (*SSU0892-0893*) were higher expressed. Accordingly, the genes for the degradation of these carbohydrate sources to glycolytic intermediates *SSU0890-0899* for lactose (tagatose pathway) and *SSU0353-0354* for maltose degradation were upregulated. In the exp growth phase the predicted PTS for fructose (*SSU0768*), trehalose (*SSU0217*) and glucose/mannose (*SSU1583*), and in the stat growth phase the PTS for predicted transport of mannose (*SSU0199-201*), galactose (*SSU0403-0406*), and N-acetylgalactosamines (*SSU1055-1057*) were significantly lower-expressed in strain 10Δ*flpS*. In both growth phases, no substantial changes in gene expression were observed for the majority of the genes of the central carbon metabolic routes such as glycolysis, the pentose phosphate pathway (PPP), the fragmentary tricarboxylic acid cycle (TCA), and genes needed for homolactic and mixed-acid fermentation of *S. suis*. Only *gapA* and *gpmA* (both glycolysis) in the exp growth phase, and *pflBD* and *adhE* (both mixed acid fermentation) in both growth phases were differentially expressed. The genes related to amino acid biosynthesis were not differentially expressed in strain 10Δ*flpS*. The attenuation of arginine degradation by the affected *arcABC* expression results in losses of ATP and carbamoyl-phosphate which is generally produced by this pathway. Carbamoyl-phosphate is needed for pyrimidine biosynthesis but can also be synthesized from glutamine by *carAB*, and in line with the attenuated *arcABC* expression, these genes and several genes of pyrimidine biosynthesis (*pyrABEFGPR*, *deoA*, *cdd*) were more highly expressed in strain 10Δ*flpS*. Since *S. suis* strain 10 is auxotrophic for glutamine, the higher demand of glutamine for driving the reaction catalyzed by the *carAB* gene product may be satisfied by the upregulation of different glutamine transporters. Accordingly, the glutamine transporter genes *glnQ1*, *glnQ4*, *glnQ5* were more highly expressed in the mutant.

We assumed that a FNR/Flp regulon may overlap with a regulon of related global regulators Crp/CcpA. Therefore, we compared the FlpS regulon with a previously published CcpA regulon which comprises not only several genes of the central carbon metabolism but also virulence-associated genes [34]. We observed in the exp growth phase of strain 10Δ*flpS* and 10Δ*ccpA* a group of genes whose expression was affected in both mutants (Figure 1C). These were the genes for PTS systems (*SSU0215-SSU0217*, *SSU1308-1310*), a phosphoglucomutase (*pgmA*), unknown membrane protein complexes (*SSU1070-SSU1073*, *SSU1924-1928*), and stress-related genes (*hrcA*, *grpE*, *dnaJK*). However, most of them displayed opposite regulation. In the stat growth phase there was a high number of similarly regulated genes (*n* = 70). Among them were genes encoding for ribosomal proteins, PTS systems (*SSU0198-SSU0199*, *SSU0402-0407*), sugar conversions (*SSU0999-1001*, *SSU1915*) and ABC transporters (*SSU1194*, *SSU1233*, *SSU1372*, *SSU1574-1575*, *SSU1851-1853*). Genes whose expression was exclusively affected in the FlpS mutant could be assigned to nucleotide metabolism (*SSU0311*, *SSU0734*, *SSU1823*) and ABC transporters (*SSU0114-0115*, *SSU1498-1499*, *SSU1824-1825*), whereas those in the CcpA mutant were assigned to carbohydrate transport (*SSU0165-0167*, *SSU1234*), carbohydrate conversions (*SSU0676*, *glgABP*, *SSU1171*, *SSU1230*, *SSU1214*) and metabolism (*ldh*, *eno*). The majority of genes were regulated in only one growth phase for each mutant strain, indicating that each of the knock-outs predominantly exhibit individual gene regulation. Taken together, FlpS is strongly involved in the activation of the *arcABC* operon and seems to be involved in the regulation of a selected group of metabolic genes.

### 2.2. Metabolic Characterization of the FlpS Knock-Out Strain *10ΔflpS*

**Figure 2:**
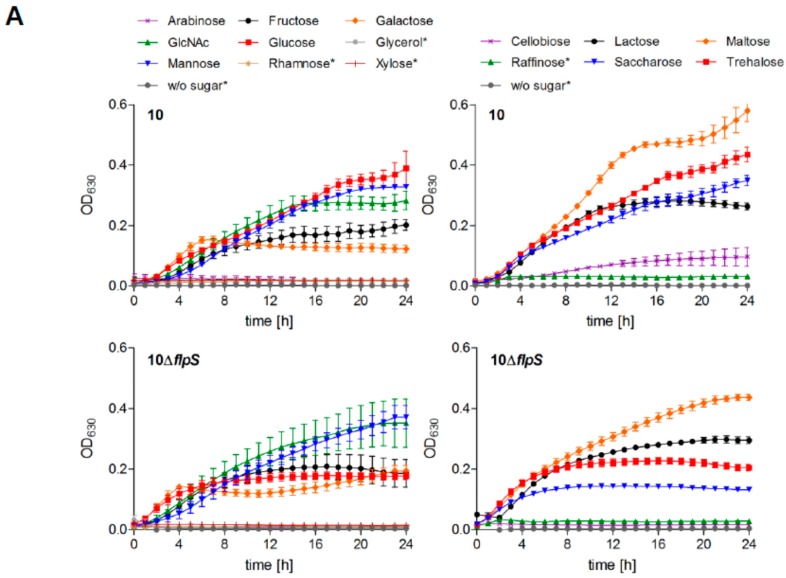

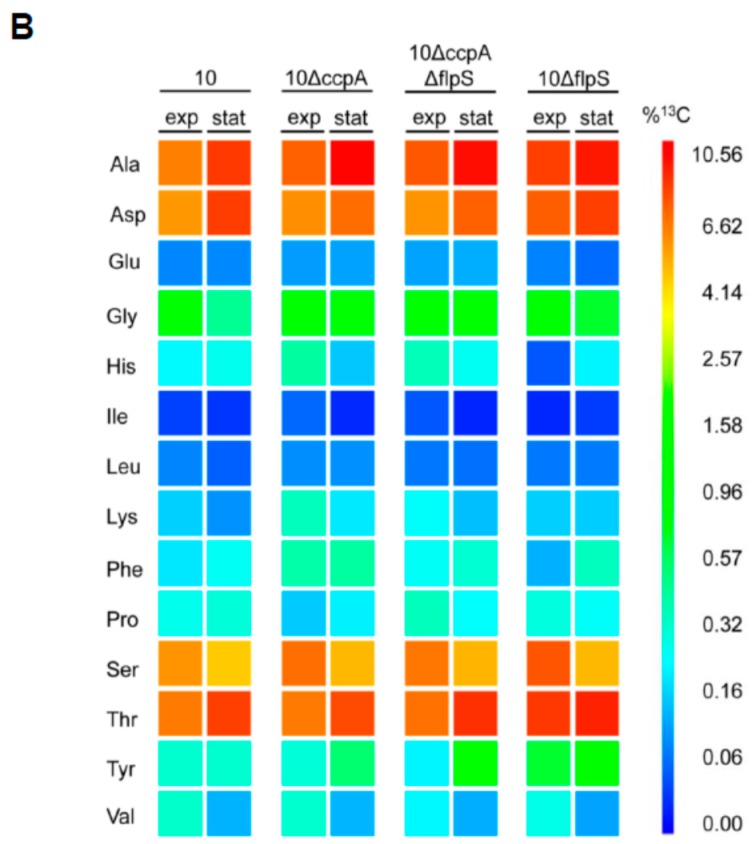
Metabolic characterization of *S. suis* strain 10Δ*flpS*. (**A**) The wild-type strain 10 and strain 10Δ*flpS* were grown in CDM medium containing 40 mM of monosaccharides (**left** panel) or di-/trisaccharides (**right** panel) indicated, and OD_630_ values were recorded at one-hour intervals automatically in a thermostatic 96-well microplate reader. Results and standard deviations are shown for three biological replicates. Carbohydrate substrates that could not be used for streptococcal growth in CDM are marked by asterisks; (**B**) Color map for the overall ^13^C excess (mol %) of labeled amino acids after growth of *S. suis* strains in the presence of [U-^13^C_6_]glucose in THB media. Notably, only overall ^13^C excesses above 0.5 mol % were considered as sufficient labeling rates. The results are shown for exp and stat grown bacteria. Mean values of two biological replicates for which MS measurements were performed in triplicate are given.

In contrast to the *arcABC*-specific regulator ArgR, FlpS seems to be additionally involved in the regulation of metabolic genes. Therefore, we analyzed if the differential expression of metabolic genes in strain 10Δ*flpS* has any relevance for metabolic activity. Since we observed that several PTS and ABC transporters were differentially expressed in strain 10Δ*flpS*, we further analyzed its ability to use different carbohydrate sources for growth in a chemically defined medium (Figure 2A). The experiments revealed reduced maximal growth of strain 10Δ*flpS* on glucose, cellobiose, and trehalose (α-α′-diglucoside). These phenotypes corresponded to the affected expression of the respective genes found by our microarray analysis. *SSU0357* and *SSU1583* presumably encode for glucose-specific PTS whereas *SSU0217* and *SSU1309* encode for trehalose- and β-glucoside PTS, respectively.

Glucose and glucose-containing di- and oligosaccharides are the major energy sources of *S. suis.* Since expression of *gapA* and *gpmA* was slightly affected in strain 10Δ*flpS*, we were interested to know if the reduced growth of strain 10Δ*flpS* on glucose might result from an altered central carbon metabolism due to changes in carbon fluxes. For this, we performed ^13^C-isotopologue profiling experiments in which ^13^C-labeled [U-^13^C_6_]glucose was supplemented for bacterial growth. In *S. suis*, metabolization of [U-^13^C_6_]glucose led to the highest molar ^13^C rates in alanine, aspartate, serine, and threonine [36]. After growth on [U-^13^C_6_]glucose we did not observe any significant differences in the overall molar ^13^C excess (Figure 2B) or the isotopologue pattern (Table S6) of these four amino acids between the wild-type strain, strain 10Δ*ccpA*, strain 10Δ*flpS* and a 10Δ*flpS*Δ*ccpA* double mutant, indicating no substantial changes in the activity of the main carbon metabolism.

### 2.3. Loss of FlpS, but not CcpA, Is Dispensable for Virulence Properties of *S. suis* in Mice Infection Models

**Figure 3:**
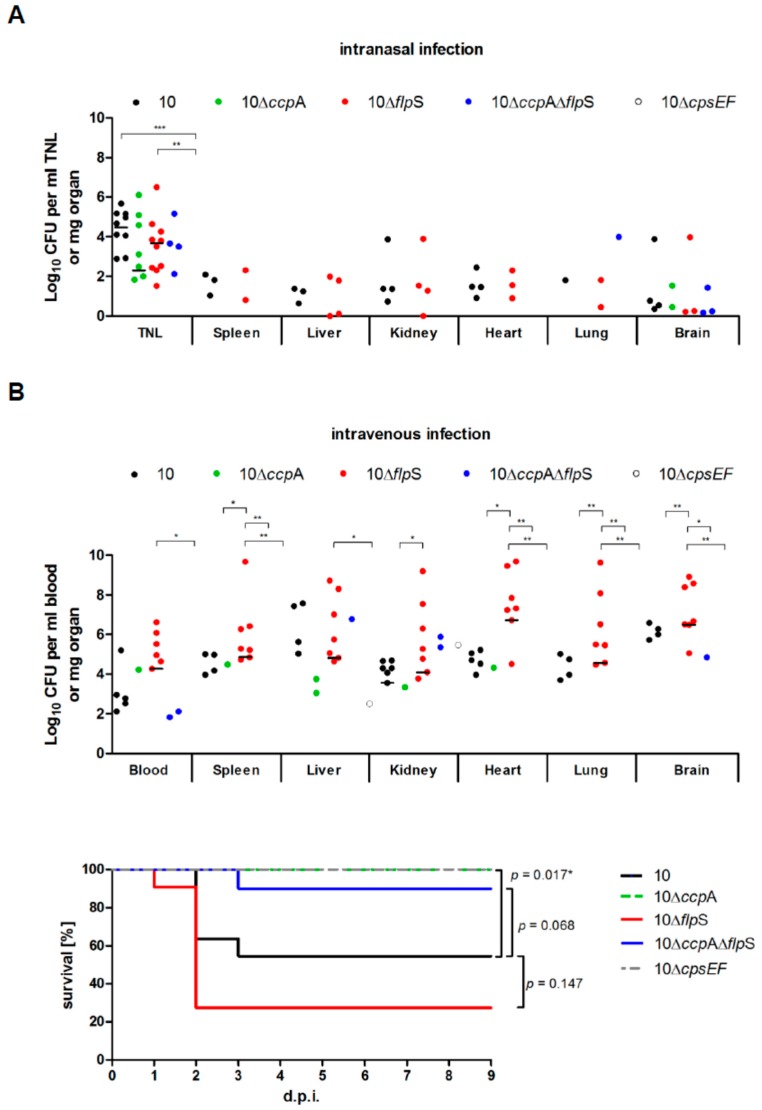
Mice infection with different *S. suis* regulator mutant strains. (**A**) Intranasal infection of mice. Specific bacterial loads of tracheonasal lavage (TNL) and indicated inner organs of mice (3 d.p.i.) intranasally infected with indicated *S. suis* strains. Each symbol represents one individual animal and medians are indicated by horizontal lines. Statistical testing was done for each TNL or organ by a Kruskal-Wallis test with a post hoc Dunn’s multiple comparisons test; (**B**) Intravenous infection of mice. The upper panel shows specific bacterial loads of blood samples and indicated inner organs of mice intravenously infected with indicated *S. suis* strains. Statistical testing was done for each blood sample or organ by a Kruskal-Wallis test with a post hoc Dunn’s multiple comparisons test. In the lower panel the respective Kaplan-Meier diagram for mortality of mice is shown. Significant difference is indicated by * with *p* < 0.05 (log-rank test).

The data above suggest that FlpS and CcpA are transcriptional regulators involved in several mostly different metabolic processes. In order to prove if a knock-out of FlpS and/or CcpA has an influence on bacterial fitness and virulence properties of *S. suis* in vivo, we conducted two different types of mice infection experiments. Firstly, we used an established intranasal infection model to study *S. suis* colonization of the murine nasopharynx [37]. As expected, none of the mice showed any clinical signs after intranasal infection, since intranasal infection leads to subclinical infection. As a read-out parameter for colonization of the upper respiratory tract, the bacterial load was determined after tracheonasal lavage (TNL). In contrast to the infection with the capsular polysaccharide-deficient strain 10Δ*cpsEF* which was not able to colonize the nasopharyngeal cavity, we observed effective colonization of the murine nasopharynx by wild-type strain 10 (in nine of 10 mice) and strain 10Δ*flpS* (in all mice) (Figure 3A). No significant differences were observed between bacterial loads in the TNL after infection with the wild-type strain 10, strain 10Δ*flpS*, strain 10Δ*ccpA*Δ*flpS* and 10Δ*ccpA*.

Secondly, we performed intravenous infection of mice. As shown by the Kaplan-Meier diagrams for mortality (Figure 3B), strain 10Δ*flpS* was as virulent as the wild-type strain 10, whereas strains 10Δ*ccpA* and 10Δ*cpsEF* were strongly attenuated. Furthermore, the significant difference between strain 10Δ*flpS* and strain 10Δ*ccpA*Δ*flpS* indicates that the phenotype of the latter is dominated by the knock-out of *ccpA*. In addition, we observed no significant differences between the bacterial loads in the inner organs after the infection with wild-type strain 10 and strain 10Δ*flpS*. However, bacterial loads in systemic organs were, in most cases, significantly higher in mice infected with strain 10Δ*flpS* in comparison to strains 10Δ*ccpA* and 10Δ*cpsEF.* Taken together, results from both the intranasal as well as the intravenous infection model indicated that the loss of CcpA but not FlpS affects the virulence properties of *S. suis* in mice.

### 2.4. FlpS is an Oxygen-Sensing Regulator and Essential for *arcABC* Operon Expression In Vitro

**Figure 4:**
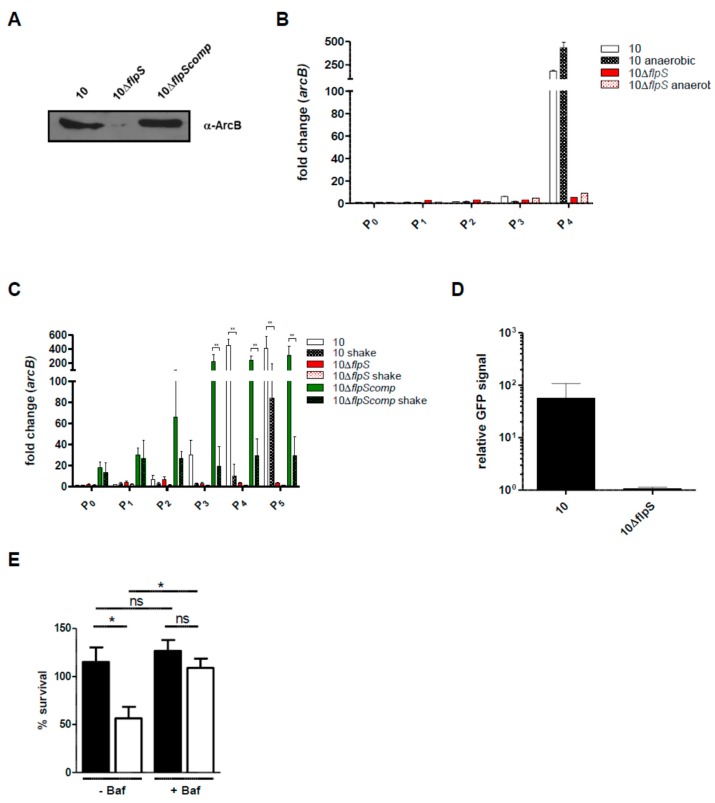
FlpS is essential for *arcABC* operon expression and fitness of *S. suis*. (**A**) Immunoblot analyses of whole-cell lysates of *S. suis* strains grown to stat phase in THB medium under anaerobic conditions. Immunoblot for wild-type strain 10, strain 10Δ*flpS* and complemented mutant strain 10Δ*flpScomp* probed with polyclonal antisera raised against recombinant ArcB; (**B**) Real-time qRT-PCR experiments of *S. suis* strain 10 and strain 10Δ*flpS* grown under standard batch and anaerobic conditions. Fold changes in relative *arcB* transcript levels were shown for a time kinetic as described in Materials and Methods. Results for two independent experiments are depicted; (**C**) Real-time qRT-PCR experiments of *S. suis* strain 10, strain 10Δ*flpS* and strain 10Δ*flpScomp* grown under batch and shaking (shake) conditions. Fold changes in relative *arcB* transcript levels were shown for a time kinetic as described in Materials and Methods. Data from three biological replicates and are shown as means ± SEM. Statistical analysis was performed using one-way ANOVA followed by a post-Tukey test (**, *p* < 0.01); (**D**) GFP reporter assay. Reporter plasmids carrying the GFP under control of the *arcABC* promoter were transformed in *S. suis* wild-type strain 10 and strain 10Δ*flpS*. Bars represent the relative fluorescence units (RFU) after normalization to the values obtained for strain 10 carrying the promoterless *gfp* construct (10::*gfp*). Experiments were carried out in triplicate and repeated twice; (**E**) Intracellular survival of the unencapsulated strain 10∆*cpsEF* (**black** bars) and its *flpS* mutant strain 10∆*cpsEF*∆*flpS* (**white** bars) in HEp-2 cells. HEp-2 cells were either treated with 200 nM bafilomycin (+Baf) for 1 h before infection to inhibit endosomal acidification or left untreated (−Baf). Results are given as percentage of intracellular bacterial survival after 2 h. Data represent means and standard deviation of two independent experiments performed in duplicates. Results were considered statistically significant with *p* < 0.05 in a two-tailed *t*-test, as indicated by asterisks.

The activity of FNR and FNR-like regulators is tightly controlled by the redox state in bacteria [14]. In *S. suis*, the activity of ArcABC is subjected to CCR and strongly activated at the stat growth phase in which free glucose is consumed [38]. We have previously shown that the activity of the arginine deiminase in *S. suis* is induced under anaerobic conditions [23]. Our microarray experiments revealed FlpS as a regulator of the *arcABC* operon since its expression was attenuated in the stat growth phase of strain 10Δ*flpS*. Therefore, we selected the *arcABC* operon as a read out to dissect FlpS activation in more detail. To show the relevance of FlpS for anaerobic expression of the ADS, we performed Western blot analysis with cell lysates of the wild-type strain 10, strain 10Δ*flpS* and a complemented mutant strain (10Δ*flpScomp*) grown anaerobically to stat growth phase. As shown in Figure 4A, ArcB expression was not detectable in strain 10Δ*flpS* whereas the wild-type expression level was restored in strain 10Δ*flpScomp* in which the *flpS* gene was provided in *trans*. The data indicated that FlpS is essential for ArcABC expression under anaerobic growth conditions. Following, we performed qRT-PCR analyses of *arcABC* operon mRNA from *S. suis* grown aerobically or anaerobically. This data confirmed that FlpS is essential for *arcABC* operon expression under aerobic and anaerobic condition (Figure 4B,C). The expression of the *arcABC* operon of the wild-type strain was induced at standard batch and anaerobic conditions as soon as the repressing effect of CCR ceased (Figure 4B). Remarkably, higher levels of expression were observed in anaerobically grown cultures when compared to batch cultures, suggesting that more active FlpS is present under anaerobic conditions. To test the influence of oxygen on FlpS activity, we increased the levels of oxygen in the aerobic growth experiments by intensive shaking of the *S. suis* cultures. The data shown in Figure 4C demonstrate that induction of *arcABC* expression was dependent on FlpS, even if the culture was shaken. However, compared to the time kinetic of the batch cultures, *arcABC* operon induction was delayed in shaking cultures, indicating that higher oxygen tension reduces FlpS activity. To further elucidate the regulatory function of FlpS in *arcABC* operon expression, we performed GFP reporter studies. For this, the wild-type strain 10 and strain 10Δ*flpS* were transformed with a plasmid carrying a *gfp* gene under transcriptional control of the *arcABC* promoter. Bacteria were grown in a minimal medium to the stationary growth phase and *gfp* activity was then determined by fluorescence measurement (Figure 4D). In contrast to the wild-type construct 10::*arcA-gfp* which is able to induce *gfp* expression, almost no *gfp* activity could be detected in strain 10Δ*flpS::arcA-gfp*, indicating that FlpS is essential for reporter gene activity. Taken together, these data confirm that FlpS is essential for *arcABC* operon expression and they provide evidence that FlpS activity is regulated by the environmental oxygen concentration.

We have previously shown that the *arcABC* operon contributes to the survival of *S. suis* in epithelial cells. For neutralization of endosomal acidification, ADS activity is important because it generates ammonia and ATP which can be used by the bacterial F_1_F_o_-ATPase to actively extrude protons. Since FlpS is essential for *arcABC* operon expression, we performed infection experiments with HEp-2 epithelial cells. We performed these experiments with a *flpS*-deficient strain in a non-encapsulated background (strain 10Δ*cpsEF*Δ*flpS*), as the polysaccharide capsule inhibits *S. suis* uptake by epithelial cells. As shown in Figure 4E, strain 10Δ*cpsEF* was able to survive and multiply intracellularly, whereas significantly lower survival rates were recorded for strain 10Δ*cpsEF*Δ*flpS*. To analyze whether the reduced survival of the *flpS*-deficient strain correlated with its inability to prevent acidification, due to defective *arcABC* operon expression, HEp-2 cells were treated with bafilomycin to inhibit endosomal acidification before infection. Compared with the infection of untreated cells, the pretreatment of the cells with bafilomycin significantly increased the survival rate of strain 10Δ*cpsEF*Δ*flpS*. These data suggest that despite having minor relevance in *S. suis* mice colonization and infection, FlpS contributes to *S. suis* resistance against endosomal acidification.

## 3. Discussion

In Gram-positive bacteria, catabolite repression can be linked to the activity of the CcpA regulator, which was characterized previously in *S. suis* by us and others [34,39]. FNR-like proteins (FLP) are found in Gram-positive bacteria and have been shown to be involved in oxygen-regulated gene expression [14,40]. However, knowledge about Flp proteins in Gram-positive bacteria and especially in *S. suis* is very limited.

By an in silico homology search, we located the gene encoding for an FNR-like protein of *S. suis* (FlpS) closely upstream of the *arcABC* operon [23]. In the present study we generated a *flp*S knock-out mutant strain and analyzed its gene expression in exp and stat growth phases in a complex medium. These analyses showed that *flp*S expression was essential for *arcABC* operon expression. Additionally, depletion of FlpS considerably influenced the expression level of multiple other genes in both growth phases. The higher and lower expression of genes suggested a repressing and activating regulatory role for FlpS. However, many other transcription factor genes were influenced (exp, *n* = 27; stat, *n* = 24) in the *flp*S mutant, so their contributions to these expression profiles have to be considered. On the other hand, the strongly increasing number of lower-expressed genes from the exp to stat growth phase might suggest that FlpS is an activator of gene expression and that FlpS itself is activated by the changing environment during bacterial growth. The majority of differentially expressed genes in both growth phases of strain 10Δ*flpS* were associated with carbohydrate transport/metabolism and nucleotide transport/metabolism. In Gram-negative bacteria (e.g., *E. coli*), FNR and CRP can regulate the expression of overlapping regulons of target genes in response to environmental (oxygen/redox) or metabolic stimuli (carbon source), respectively [14]. Therefore, we assumed that FNR/Flp regulation may overlap with the regulation of the related regulator CcpA. Hence, we compared the FlpS regulon with a previously published CcpA regulon [34]. These data revealed a group of genes whose expression was affected in both mutants, especially in the exp growth phase. However, most of them displayed opposite regulation in strains 10Δ*flpS* and 10Δ*ccpA*. Nevertheless, this comparison enabled us to dissect the metabolic role of FlpS more precisely. The genes whose expression was exclusively affected in the CcpA mutant assigned to carbohydrate transport, carbohydrate conversions and metabolism reflect the sovereign role of CcpA in CCR. In contrast, the genes exclusively affected in the FlpS mutant assigned to nucleotide metabolism and ABC transporters indicate a role of FlpS in the regulation of these processes. An influence on glycolysis by FlpS might have been deduced from the differential expression of *gapA* and *gpmA* in the mutant but this could not be confirmed by the isotopologue pattern of alanine, an amino acid which is directly synthesized from the end product of glycolysis. Furthermore, in correlation with the isotopologue data, we did not observe any significant changes in amino acid preferences of strain 10Δ*flpS* in growth experiments in which we subsequently omitted each of the main 20 amino acids in CDM media (data not shown). Overall, these experiments indicate that under our experimental conditions, the impact of FlpS in *S. suis* metabolism is rather low. This is further underlined by the results of our mice infection experiments. In contrast to the CcpA mutant whose attenuation is presumably driven by alteration virulence-associated factor expression [38], the *flpS* mutant was as virulent as the wild-type strain.

The FlpS gene is located closely upstream of the *arcABC* operon and our data indicate that FlpS is essential for *arcABC* expression. We have previously shown that *arcABC* operon expression in *S. suis* depends essentially on the regulator ArgR [33] and is subjected to CCR, however, not as a direct target of CcpA [34]. The CCR of the *arcABC* operon explains its exclusive expression at the late stage of bacterial growth when glucose is almost consumed. FlpS does not influence *argR* expression; therefore, the loss of *arcABC* operon expression in the absence of ArgR [33] or FlpS indicates that both regulators cannot compensate for each other, and that they may synergistically induce the *arcABC* operon, as also proposed for other bacteria [20,25,26,41]. Similar to ArgR, the regulatory activity of FlpS can be restricted to the promoter region of the *arcABC* operon; nevertheless, future studies will have to show whether FlpS directly regulates *arcABC* operon expression by binding to the promoter. FlpS shows homologies to the FNR-like regulators FLP of *S. gordonii*, ArcR of *Bacillus licheniformis*, and ArcR of *Staphylococcus aureus* [23], all of which have been shown to regulate the arginine deiminase genes in the respective species under anaerobic conditions [13,20,26]. Accordingly, we found that strain 10Δ*flpS* was not able to induce *arcABC* expression when grown under anaerobic conditions. The fact that FlpS is also essential for *arcABC* expression in *S. suis* grown under aerobic conditions denotes FlpS as a regulator whose activity is dependent on the redox state of the cell which might be influenced by free oxygen or by an as-yet-unknown mechanism.

Our data provide evidence for an involvement of FlpS in oxygen- and/or redox-related responses of *S. suis.* Thus, the FNR regulator in *E. coli* is known to regulate the expression of genes under anaerobic conditions, including fumarate reductase, nitrate and nitrite reductase, and cytochrome d oxidase genes, all of which are enzymes involved in processes of the metabolic transition between aerobic and anaerobic growth [42,43,44]. The FlpS regulon of *S. suis* comprises genes whose expression was influenced in *S. intermedius* and *S. mutans* when grown under anaerobic conditions [45,46]. Similar to *S. intermedius*, we found upregulation of glycolytic genes, pyruvate-formate lyase, alcohol dehydrogenase, the arginine deaminase pathway and the nucleotide synthesis/salvage pathways [45]. In addition, similar to the response of *S. intermedius* and *S. mutans* [45,46], we found altered expression of PTS and ABC transporters for utilization of C5 and C6 sugars (e.g., glucose, trehalose) in strain 10Δ*flpS*. Furthermore, among the oxidative stress–related genes, the gene of the Dps-like peroxide resistance protein (Dpr) was differentially expressed in strain 10Δ*flpS*. Lastly, even the enhanced *arcABC* expression when *S. suis* was grown under anaerobic conditions and the delay in *arcABC* expression when the oxygen tension in the culture medium was increased indicate the oxygen-dependent activity of FlpS.

FlpS of *S. suis* carries two cysteine residues at the N-terminus which might contribute to iron-sulfur cluster binding. After the exchange of one cysteine residue by a serine at position 129 (C129S), we did not observe any differences in *arcABC* expression in comparison to the complemented mutant strain carrying the wild-type *flpS* allele (data not shown). This observation, however, does not exclude oxygen-dependent regulation of FlpS since FlpS of *S. suis*, similar to the Flp protein of *Lactobacillus casei*, might be active in the absence of iron-sulfur cluster formation [14]. On the other hand, FlpS activity might require binding of an oxygen-labile iron-sulfur cluster by N-terminal non-cysteinyl residues, which has been described for the FNR-like protein FlpA of *Lactococcus lactis* [15]. Further studies are needed to functionally dissect the mechanism of redox regulation of *S. suis* FlpS.

A particular role of FlpS for virulence has not been shown yet. Like other pathogenic streptococci, *S. suis* is able to infect and survive in epithelial cells. This feature is largely associated with a functional ADS [23,33,47]. Our data show that FlpS contributes to intracellular survival of *S. suis*. The reduced survival of the *flp*S mutant in HEp-2 cells might be explainable by its absolute necessity for *arcABC* expression. In contrast to the cell culture infections, the *flpS* knock-out seemed not to affect *S. suis* colonization and survival in two experimental mouse models. Rather, results from intravenously infected mice suggest better survival of strain 10Δ*flp*S than the wild-type strain 10 in the inner organs. However, these data were not statistically significant and in the CcpA/FlpS double mutant, the knock-out of *flpS* could not compensate the loss of CcpA. Overall, these data suggest that the phenotype of strain 10Δ*flp*S is not advantageous when *S. suis* has to counteract a complex immune response or has to metabolically adapt to the in vivo environment in mice. The discrepancy between in vitro and in vivo data emphasizes that the role of FlpS in infection might be limited to a particular niche in its primary host, the pig, where the bacterium is exposed to specific redox conditions. This hypothesis, however, awaits further elucidation.

## 4. Materials and Methods

### 4.1. Bacterial Strains and Growth Conditions

The highly virulent *S. suis* serotype 2 strain 10 (Smith et al., 1999) and respective mutant strains used in this study are listed in Table S1. *S. suis* was cultured at 37 °C on Columbia blood agar base (Oxoid) containing 6% (*v*/*v*) sheep blood or horse blood supplemented with spectinomycin (100 μg/mL), and/or erythromycin (2 μg/mL) if necessary. If not stated otherwise, broth cultures of *S. suis* were obtained by overnight culturing in Todd–Hewitt Broth (THB, Becton Dickinson Diagnostics). On the next day overnight culture were diluted in prewarmed THB medium to an OD_600_ = 0.02 for growth experiments. Growth experiments (supplementation of different carbohydrate substrates; omitting single amino acids) in chemically defined medium (CDM) were performed in a 96-well microplate reader as previously described [36].

### 4.2. Mutagenesis of *S. suis*

The inactivation of the *flpS* gene (SSU0579) was done by insertion mutagenesis in the parental *S. suis* strain 10, the capsular polysaccharide deficient strain 10∆*cpsEF::spc^R^* (10∆*cpsEF*) or the *ccpA* deficient strain 10∆*ccpA::Em^R^* (10∆*ccpA*). For this, two mutagenesis plasmids each carrying a different antibiotic resistance cassette were constructed. In both cases a 1509-nucleotide PCR fragment comprising the *flpS* locus was amplified by PCR from chromosomal DNA of *S. suis* strain 10 with oligonucleotide primers AdR1 and AdR2. The PCR product was subcloned in the *E. coli* cloning vector pGEM-T Easy (Promega) to create pGEMAdR. Then, plasmid pGEMAdR was linearized by *SnaBI* and ligated to a *EcoRV-SmaI* fragment derived from vector pICspc containing the spectinomycin resistance cassette [48], resulting in pGEMFlpSspc. By the insertion of the antibiotic resistance cassette the *flpS* gene becomes disrupted. In order to construct a erythromycin resistance conferring mutagenesis plasmid, linearized pGEMAdR was ligated to the *PvuII* fragment derived containing the erythromycin resistance cassette from vector pICerm [49], resulting in pGEMFlpSermR. Electrotransformation of the above listed *S. suis* strains with pGEMFlpSspc or pGEMFlpSermR (see also Table S1) was performed as previously described [23]. Successful mutation was verified by PCR (data not shown) and Northern Blot analysis [38].

### 4.3. Episomal Complementation of S. suis 10∆flpS

For complementation experiments, *flpS* was amplified from *S. suis* DNA with the primer pair flpSagg-SalI and flpSend-PstI and subsequently cloned into the *S. suis-E. coli* shuttle vector pORI23 [50]. Thereby, the *flpS* gene was set under control of a constitutive P23 lactococcal promoter and the original ribosome binding-site (RBS) ‘ggagt’ in this promoter was changed to the optimal RBS of gram-positive bacteria ‘ggagg’, finally yielding plasmid pORI23-flpS. For the site directed exchange of a cysteine to a serine in the amino acid sequence of FlpS at position 129 we used pORI23-flpS as a template and applied primer pair FlpS_C129S_for/FlpS_C129S_rev according to the manual instructions of the QuikChange^®^ Site-Directed Mutagenesis Kit (Stratagene). The resulting plasmid pORI23-flpS(C129S) and also pORI23-flpS were then introduced into electro-competent *S. suis* strain 10∆*flpS::spc^R^*. Transformants were confirmed by PCR and DNA sequencing.

### 4.4. Microarray Analysis

Comparison of gene expression between the wild-type and *flpS*-deficient strain was done by microarray analyses. For this, *S. suis* strain 10 and strain 10∆*flpS* were grown in THB medium to the exponential growth and early stationary growth phase, harvested and RNA extracted from bacteria as described [33]. cDNA microarray experiments and subsequent data analysis was performed as previously specified [33]. All microarray data have been submitted MIAME complied with ArrayExpress under Accession No. E-MTAB-3997. Venn diagrams were constructed with VENNY 2.1.

### 4.5. Real-Time Quantitative RT-PCR (qRT-PCR)

For qRT-PCR experiments bacteria were grown in THB media under anaerobic conditions (anaerobic chamber (Don Whitely Scientific; atmosphere: 5% CO_2_, 10% H_2_, 85% N_2_), standard batch conditions, or shaking conditions (200 rpm). Bacteria were harvested at the exponential growth phase (OD_600_ = 0.3; P_0_) and then hourly after this time point as indicated by serially increasing numbers (e.g., P_1_, one hour after P_0_). After RNA extraction, reverse transcriptase reactions followed by quantitative PCR (qPCR), and evaluation of qPCR were done as described [34]. Relative target gene transcript levels were first normalized to *gyrB* transcript levels and then expressed as 2^−ΔΔCT^ values with regard to time point P_0_ of the wild-type strain [51].

### 4.6. Western Blot Analysis

Bacteria were anaerobically grown overnight in THB medium, on the next day diluted 1:15 in THB and further incubated under anaerobic conditions. Eight hours later bacteria were harvested, lysed and an ArcB-specific immunoblot was performed as described [33].

### 4.7. GFP Reporter Studies

GFP reporter experiments were performed with the wild-type strain carrying a promoter less GFP construct (10::*gfp*), the wild-type strain carrying a transcriptional fusion of the *arcABC* promoter to the *gfp* gene (10::*arcA-gfp*) [33], and the *flpS* mutant strain carrying the *arcABC-gfp* element (10Δ*flpS::arcA-gfp*). Strain 10Δ*flpS::arcA-gfp* was constructed by electroporation of plasmid pGA14-Parc187-gfp into strain 10Δ*flpS*::Erm^R^ and GFP reporter experiments were conducted as described [34].

### 4.8. Isotopologue Profiling

Bacteria were grown in THB medium supplemented with 10 mM [U-^13^C_6_]glucose to the exponential and early stationary growth phase, were then harvested and processed as specified recently [36].

### 4.9. Intracellular Survival in HEp-2 Cells

The ability of the unencapsulated strain 10 (10∆*cpsEF*) and its *flpS* mutant strain (10∆*cpsEF*∆*flpS*) to survive in untreated for bafilomycin (to inhibit endosomal acidification) treated HEp-2 cells was determined as described previously [33].

### 4.10. Experimental Infection of Mice

The animal experiments of the present study were approved by the Committee on Animal Experiments of the Lower Saxonian State Office for Consumer Protection and Food Safety (Niedersächsisches Landesamt für Verbraucherschutz und Lebensmittelsicherheit, LAVES; approval number 33.12-42502-04-12/0935). Infection experiments were in strict accordance with the German regulations of the Society for Laboratory Animal Science (GV-SOLAS) and the European Health Law of the Federation of Laboratory Animal Science Associations (FELASA). Furthermore, all experiments were planned to fulfill ‘3R’ rules on reduction, refinement and replacement of animal infection experiments.

For all experimental *S. suis* infections five- to six-week-old female C57BL/6J WT mice (Charles River WIGA, Sulzfeld, Germany) were infected with *S. suis* strains grown to the exponential growth phase (OD_600_ = 0.2). Intranasal infection of mice was exactly done as previously described with the exception that a higher dose was applied [19]. Briefly, after acetic acid predisposition mice were intranasally infected with a dose of approximately 1 × 10^9^ CFU. Infection experiments were done in groups of five mice and repeated once (in total 10 mice per *S. suis* strain). After infection, mice were constantly monitored for clinical signs according to an established clinical score matrix [37] but sacrificed three days post infection (d.p.i) for comparative histological and bacteriological screenings. The determination of the bacterial load in tracheonasal lavages (TNL) and indicated inner organs was performed as described [19,37]. In case of the intravenous infection model, mice were infected with approximately 5 × 10^8^ CFU in 100 μL of PBS via the tail vein. Infected mice were sacrificed 9 d.p.i if they have not been euthanized earlier due to animal welfare reasons as also judged by the investigated clinical score matrix [37]. Bacteriological and histological screenings of mice tissues were done as described earlier [19,37]. For both infection models differences in bacterial loads in TNL, blood, or organ samples were analyzed by a Kruskal-Wallis test with a post hoc Dunn’s multiple comparisons test. Statistical analysis of Kaplan-Meier diagrams was conducted with the log-rank test.

## Supplementary Materials

The following are available online at: http://www.mdpi.com/2076-0817/5/3/51/s1. Figure S1: Growth of *S. suis* strains and verification of the *flpS* knock-out. (A) Growth of wild-type strain 10, strain 10Δ*flpS* and strain 10Δ*flpScomp* in THB under batch conditions; (B) Northern blot analysis of wild-type strain 10 and strain 10Δ*flpS* along the growth phase. The blot was incubated with a [α-^32^P]dCTP labeled *flpS* probe. The ethidium bromide gel showing prominent 16S and 23S rRNA bands serves as a loading control, Table S1: Bacterial strains, plasmids, and oligonucleotides used in this study, Table S2: Validation of microarray data by RT-qPCR, Table S3: Functional COG classification of differentially expressed genes deduced from comparative microarray analyses, Table S4: Overview of significantly higher- and lower-expressed genes as determined by comparative DNA microarray analysis of the *S. suis* wild-type strain 10 and 10Δ*flpS* during exponential growth, Table S5: Overview of significantly higher- and lower-expressed genes as determined by comparative DNA microarray analysis of the *S. suis* wild-type strain 10 and 10Δ*flpS* during stationary growth, Table S6: ^13^C-excess (mol %) of amino acids in experiments with *S. suis* strain 10, 10Δ*flpS*, 10Δ*ccpA*, and 10Δ*ccpA*Δ*flpS* cultivated in THB media supplemented with 10 mM [U-^13^C_6_]glucose.

## Abbreviations

The following abbreviations are used in this manuscript:

ADS	arginine deiminase system
CcpA	catabolite control protein A
exp	exponential
FlpS	FNR-like protein of *S. suis*
stat	stationary
