# Supplementary figures and images for: FlpS, the FNR-Like Protein of Streptococcus suis Is an Essential, Oxygen-Sensing Activator of the Arginine Deiminase System
Source: Pathogens. 2016 Jul 21;5(3):51. doi: 10.3390/pathogens5030051 (PMC5039431; doi:10.3390/pathogens5030051)

Supplementary Figure S1

A

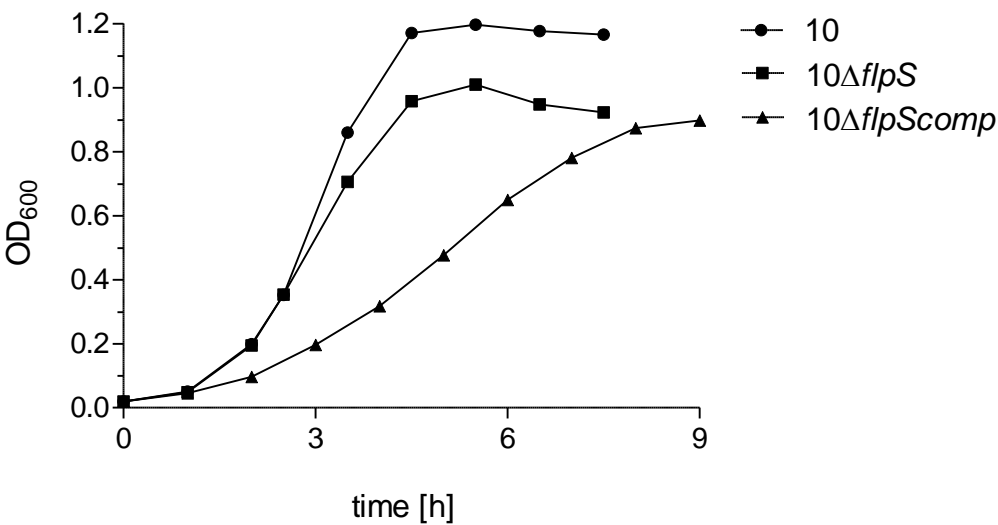

B

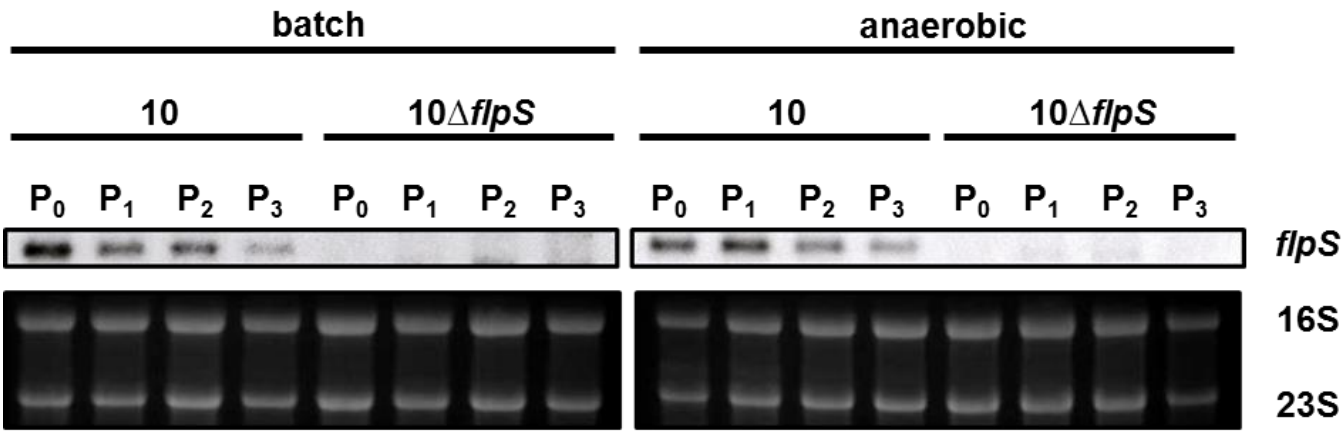

Supplement: Supplementary file 1 [file pathogens-05-00051-s001.zip › pathogens-05-00051-Supplementary/Supplementary Figure S1.pdf]
